# 

**Published:** 1889-08

**Authors:** 


RECEIPTED FOR 1889.--Drs. T J Jones, J J Poor, to may; H H Whitaker to June, C J Carter, to July. For 1888. I T Young. Robt. Sellers, Jas. Whitlock, E L Haney, R C Midley, A L Hawthorne, B F Pellow, R E Suttles, T J Wilkin, F F Sheppard.

